# Comment on “Pregnancy Screening before Diagnostic Radiography in Emergency Department; an Educational Review” 

**Published:** 2019-04-06

**Authors:** Harmen Bijwaard, Fleur Wit

**Affiliations:** 1 Medical Technology Research Group, Inholland University of Applied Sciences, Haarlem, The Netherlands; 2 Centre for Safety, National Institute for Public Health and the Environment, Bilthoven, The Netherlands

With great interest we have read the paper “Pregnancy Screening before Diagnostic Radiography in Emergency Department; an Educational Review” by A.I. Abushouk et al. (1). We agree with the authors that unnecessary fetal radiation exposure should be avoided and that pregnancy screening can be a means to accomplish this. However, in their paper the authors suggest in several instances that radiological imaging during pregnancy can lead to teratogenic effects. In the Abstract it is stated: “Radiation exposure during pregnancy may have serious teratogenic effects to the fetus. Therefore, checking the pregnancy status before imaging women of child bearing age can protect against these effects.”, and in the Introduction: “Therefore, checking the pregnancy status before imaging women of child bearing age can protect against radiation teratogenic effects.” We strongly disagree with these statements: common radiological imaging will usually not give rise to fetal radiation doses high enough to lead to teratogenesis. The statements in the paper may lead to unnecessary worrying of pregnant women and it may discourage them from undergoing medically necessary radiological examinations.

“Teratogenesis” comes from the Greek words for “monster” and “producing”. It is used to describe the induction of malformations in the fetus by toxic agents. According to the International Commission on Radiological Protection (ICRP, 2000) such malformations may occur after exposure of the fetus to a radiation dose of at least 0.1 Gy or more (2). However, in many cases the fetus will receive hardly any irradiation from a radiological examination. This is the case when the fetus is outside the x-ray beam, for example during a CT-scan of the brain of the mother. In the potentially worst cases the fetus is located directly in the x-ray beam, such as during pelvic examinations. A pelvic x-ray will usually lead to a radiation dose to the fetus of approximately 0.001 Gy and a pelvic CT-scan may lead to a fetal radiation dose of 0.025 Gy (ICRP, 2000). Slightly higher numbers are also quoted by Abushouk et al. who write: “The usual radiation dose, delivered during plain x-ray imaging, is usually less than 0.02 Gy (2 rad), while it rises to 0.02-0.035Gy (2-3.5 rad) during computed tomography (CT). Based on these calculations, even repeated abdominal or pelvic CT imaging should pose no theoretical risk to the fetus.” However, they continue with the following statement: “However, the National Council on Radiation Protection and Measurements stated a principle entitled "As low as reasonably achievable" or "ALARA" which highlighted that no radiation exposure level is entirely free of risk and that the safety of the procedure should be evaluated in terms of beneﬁt versus risk. In 2006, the National Academy of Sciences issued a report which highlighted the link between low levels of radiation exposure and the risk of teratogenesis and cancer induction.” This statement again suggests that there exists a link between low levels of radiation exposure and the risk of teratogenesis, where in fact there is only a link between these low exposures and a slightly increased risk of cancer induction. Common radiological procedures will not lead to teratogenesis. This may only occur in the very rare case that very many (pelvic) radiological procedures are carried out or when either radiotherapy or an extensive interventional radiological procedure is carried out during which the fetus is located in the x-ray beam. 
